# MOVIS: A multi-omics software solution for multi-modal time-series clustering, embedding, and visualizing tasks

**DOI:** 10.1016/j.csbj.2022.02.012

**Published:** 2022-02-22

**Authors:** Aleksandar Anžel, Dominik Heider, Georges Hattab

**Affiliations:** Department of Mathematics and Computer Science, University of Marburg, Hans-Meerwein-Strasse 6, Marburg 35032, Hesse, Germany

**Keywords:** Time-series, Multi-omics, Visualization, Data exploration, Temporal multi-omics, Longitudinal multi-omics

## Abstract

Thanks to recent advances in sequencing and computational technologies, many researchers with biological and/or medical backgrounds are now producing multiple data sets with an embedded temporal dimension. Multi-modalities enable researchers to explore and investigate different biological and physico-chemical processes with various technologies. Motivated to explore multi-omics data and time-series multi-omics specifically, the exploration process has been hindered by the separation introduced by each omics-type. To effectively explore such temporal data sets, discover anomalies, find patterns, and better understand their intricacies, expertise in computer science and bioinformatics is required. Here we present MOVIS, a modular time-series multi-omics exploration tool with a user-friendly web interface that facilitates the data exploration of such data. It brings into equal participation each time-series omic-type for analysis and visualization. As of the time of writing, two time-series multi-omics data sets have been integrated and successfully reproduced. The resulting visualizations are task-specific, reproducible, and publication-ready. MOVIS is built on open-source software and is easily extendable to accommodate different analytical tasks. An online version of MOVIS is available under https://movis.mathematik.uni-marburg.de/ and on Docker Hub (https://hub.docker.com/r/aanzel/movis).

## Graphical abstract

**Figure:**
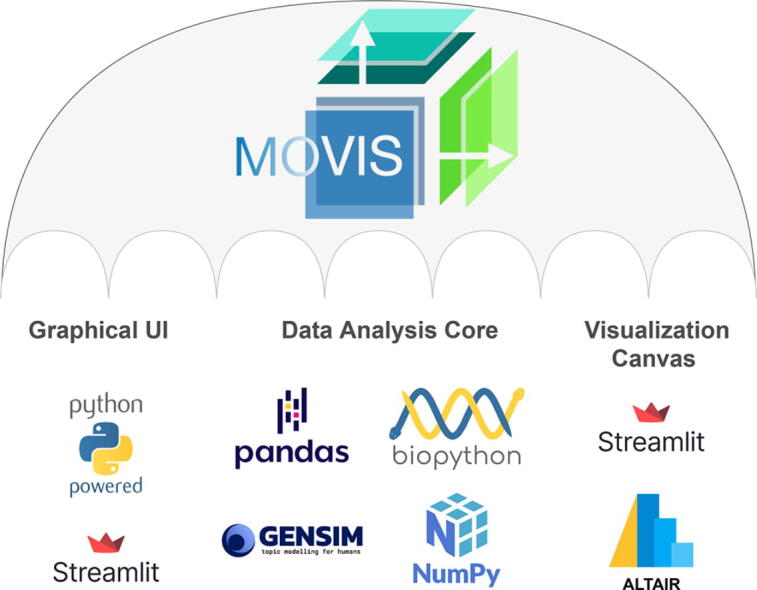

## 1 Introduction

High-throughput technologies allow us to generate large amounts of data that could be used for medical and biological research. Multi-omics data sets have been extensively used to provide new insight into certain diseases such as cardiovascular diseases [1], type 2 diabetes [2], cancer [3], and infectious diseases [4]. With the new technologies mentioned above and technical advances in computational power, data sampling could become more granular. Data sets taken at one point in time can now be easily extended by creating new data sets at other time points, providing a more detailed picture of the underlying biological phenomena. These types of time-series data sets are becoming more common and are often explored using machine learning methods [5]. Although the demand for integrative, analytical, and explorative tools for such data is high, only a handful exists, e.g., TIMEOR [6], PyIOmica [7], and Functional Heatmap [8]. Functional Heatmap was developed as a web-based tool for time-series transcriptomics data sets. Analysis results can be exported in textual format or visually thanks to data visualizations, yet only using heatmaps or parallel coordinate plots. TIMEOR was also developed as a web-based tool for defining regulated gene networks from gene-related time-series data sets using RNA-seq, and protein-DNA interaction (such as ChIP-seq [9] and CUT&RUN [10]) techniques. In contrast, PyIOmica was developed as a Python [11] library with the ability to work with different time-series omics data sets, like proteomics, metabolomics, etc. It also includes gene ontology (GO) and Kyoto Encyclopedia of Genes and Genomes (KEGG) pathway enrichment analyses. PyIOmica currently represents the most complete solution for working with time-series multi-omics data sets. Still, as a library, it does not allow domain specialists like biologists and biophysicists to conduct an exploratory data analysis. Besides that, none of these tools allow a step-wise, easy-to-follow pipeline, providing both control and freedom to explore the data and discover irregularities or trends in the data. With the challenge of multi-modal and temporal data, the analysis of multiple omics should provide multiple aspects that vary in scope and detail. For example, a side-by-side view of the outcome of each analysis integrating the same time span is necessary. To overcome these limitations and reduce the separation between each omic-type data, we developed the Multi-Omics VISualization (MOVIS) tool. MOVIS is a web-based, modular tool that enables easy exploration of time-series multi-omics data sets in a side-by-side fashion. In turn, it enables both developers and domain specialists to formulate and test their hypotheses. While the modularity of the core components enables developers to separate and recombine low-level functionalities, it supports requests for additional functionalities or omics-specific tasks. By means of modularity and using open-source libraries, it can be easily extended to accommodate new use case scenarios. To our knowledge, MOVIS is the first freely available time-series multi-omics data exploration tool and a pipeline for creating publication-ready visualizations.

## 2 Approach

**Fig. 1:**
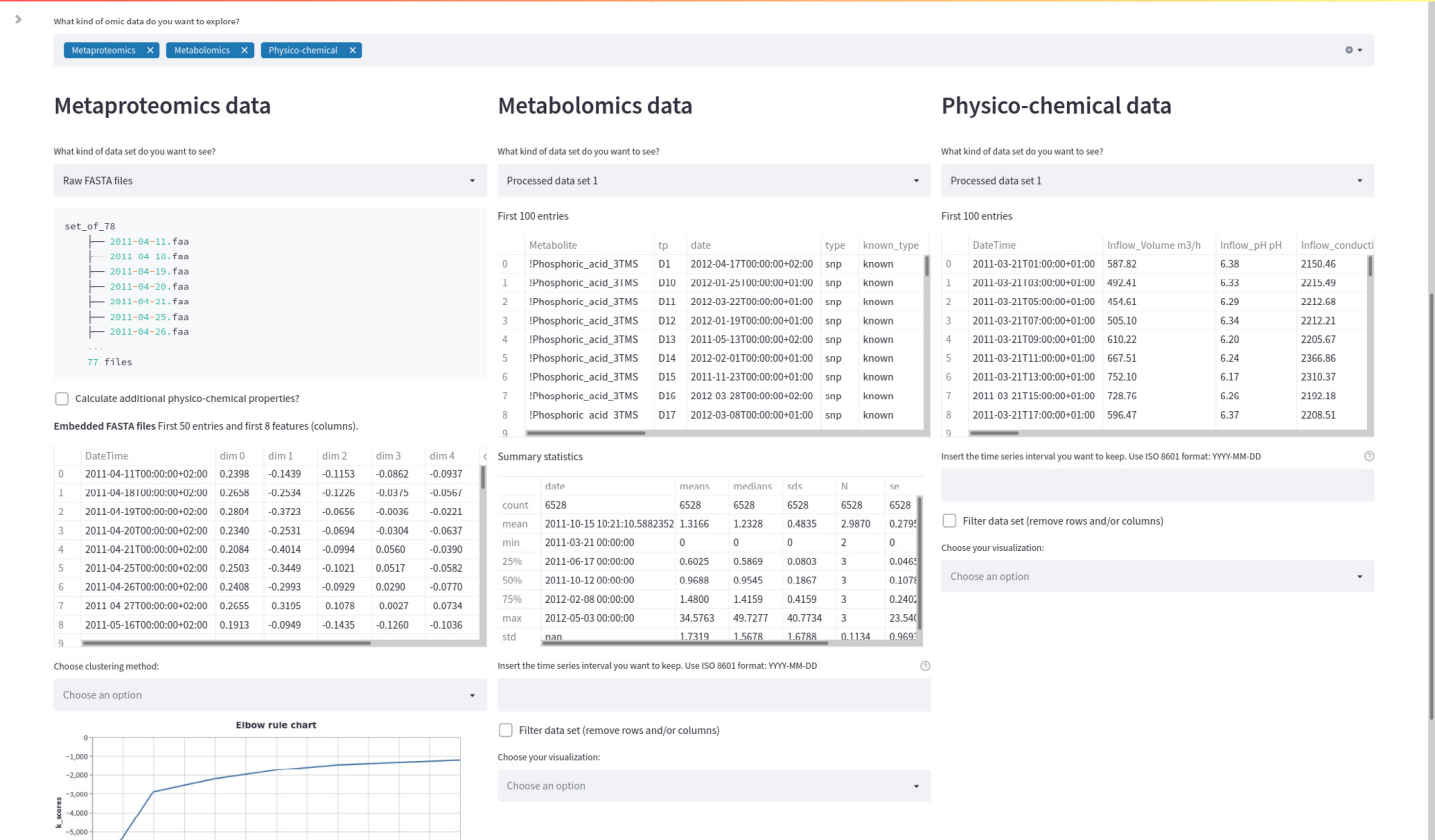
Split view of multiple omics. One of the most powerful functionalities of MOVIS is the ability to represent each omic-type in its own view space. This allows the user to inspect and explore multi-omics data sets at the same time.

**Fig. 2:**
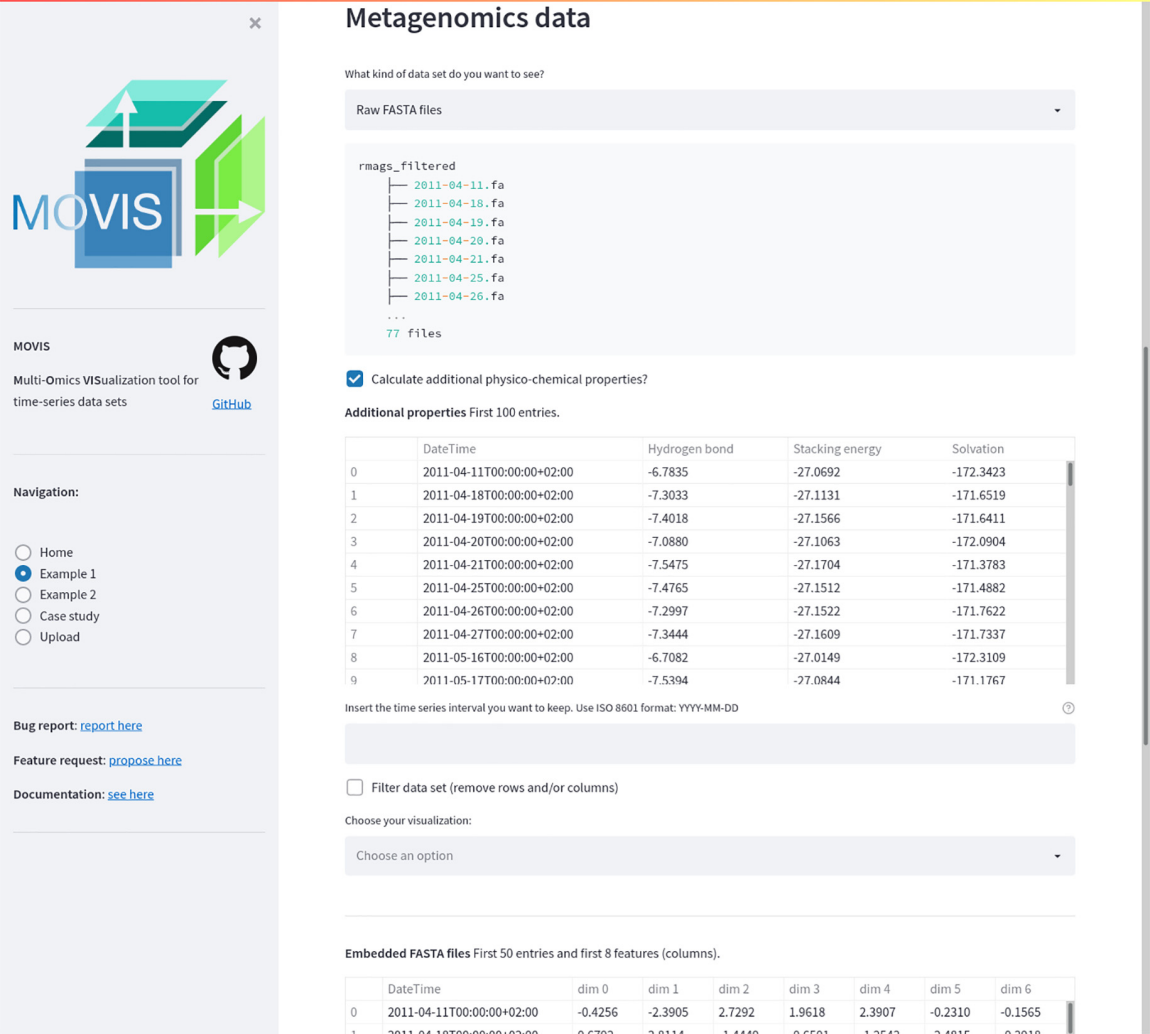
Step-wise process of the MOVIS workflow. Data exploration and visualization of each omic are divided into multiple steps. This figure shows steps (1), (2), and (3) for the metagenomics data set.

**Fig. 3:**
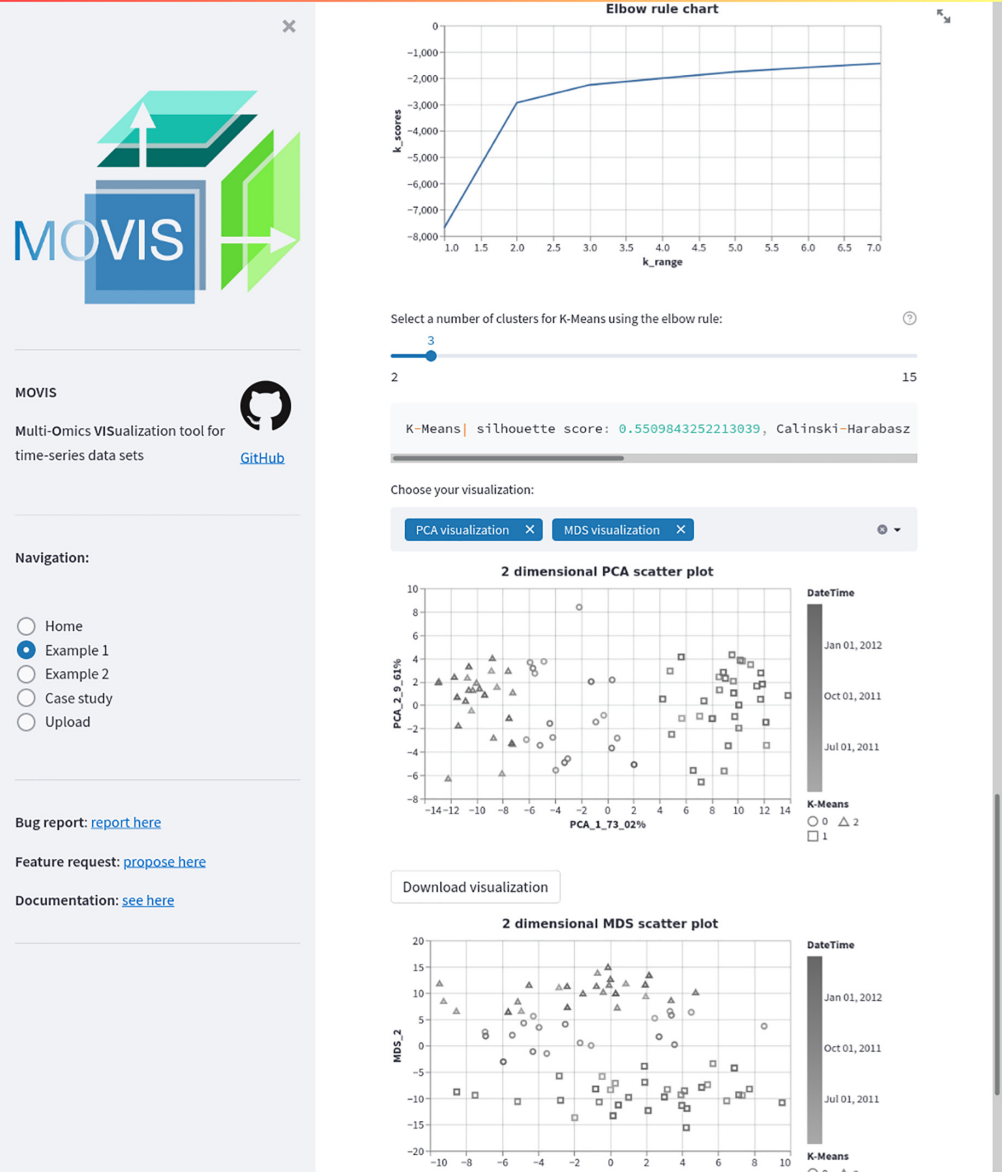
Exported visualizations for example data 1. The user has the ability to export resulting visualizations in several lossless formats with just a few mouse clicks. In this figure, we can see save buttons for the scatter-plot visualization of embedded genomics data. Similar exported publication-ready visualizations could be seen in Fig. 9.

**Fig. 4:**
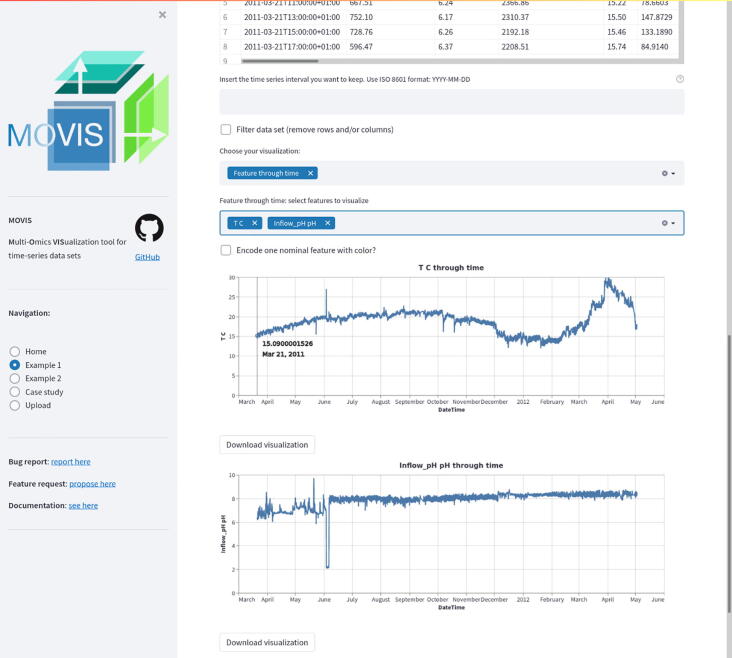
isualization canvas with multiple visualizations. Visualization canvas is the part of the UI that holds visualizations for all data sets in use. It is separated from the data exploration part of MOVIS in order to make the exploration part distraction-free and continuous. This figure also shows the interactive part of every visualization, in this case, a tooltip with additional information of the underlying data point. Further filtering is available to look at the specific time frame.

MOVIS consists of three distinct parts: (1) a graphical web interface, (2) a data analysis core, and (3) a visualization canvas. The interactive graphical web interface is built on the open-source framework Streamlit (https://streamlit.io/). The user interface (UI) allows the user to split the screen into multiple views. Each view corresponds to one of five omics (genomics, proteomics, transcriptomics, metabolomics, physico-chemical data) available to work with. This functionality is presented in Fig. 1. UI also consists of a side panel used for navigation and shows basic information about the tool. The whole workflow is divided into five well-defined parts: (1) the original data set presentation, (2) optional creation of a new data set, (3) optional data set filtering, (4) optional data clustering, and (5) data set visualization. Some steps of the workflow are shown in Fig. 2. The data analysis core is responsible for five sequential core steps: importing, sanitizing, filtering, analyzing, and visualizing all data sets. The core works with five different types of omics data. In the first core step, i.e., importing, genomics, and proteomics data sets can be provided as archived FASTA files (ZIP, TAR, etc.) or as precalculated tabular (CSV or TSV) files. For the genomics data, archived GFF, KO, and Depth-of-coverage files are also supported. The latter is available for transcriptomics as well. Metabolomics, transcriptomics, and physico-chemical data sets can be provided as tabular files. In the case of transcriptomics data, users can upload multiple tabular data sets. However, each data set must have the same set of columns with precisely the same names. MOVIS concatenates these data sets into one unified data set with one new feature (column) named *Type*. This column contains the names of all user-uploaded files. Multi-tabular functionality is provided to enable more accessible work with multiple biological and technical replicate files simultaneously, which is common when researching gene expression. The sanitizing step is dependant on the data set format provided to the core. For archived data sets, the sanitizing step consists of unpacking the archive, checking the validity of the file names, and correcting them if they do not adhere to the naming rules. We created these rules so that file names could hold standardized temporal information of each file. If a data set is of a tabular format, the sanitizing step consists of various data quality checks. In this case, temporal information must be included as a feature of the data set. The filtering step is present only for tabular data sets. It offers a way to filter a certain time-series period and remove one or more rows or columns from the data set. The data analysis step is the central and most intricate part of the data analysis core. It provides embedding and clustering functionalities, as well as creating a new physico-chemical data set for archived FASTA data sets. The visualization step is responsible for visualizing and additional filtering of the data sets. It also implements interactivity to the resulting visualizations in the form of a tooltip, brushing, and/or spanning and zooming. The visualization canvas provides a unified representational space for all created visualizations. It enables users to export the created visualization in several formats. Each visualization can be exported as a PNG or SVG file or a Vega-Lite [12] source specification. This functionality is presented in Fig. 3. Currently, MOVIS supports nine different visualizations of time-series data sets: *Correlation heatmap*, *Time heatmap*, *Multiple features parallel chart*, *Scatter-plot matrix*, *Scatter plot*, *Two features plot*, *Feature through time*, *Whisker plot*, and *Top 10 share through time*. The user could use each of the visualizations for any data set type without any restrictions. However, some basic data knowledge is advised in order to choose appropriate visualizations for certain data set types. The visualization canvas, along with multiple visualizations, is shown in Fig. 4.

## 3 Methods

MOVIS is built using Python and additional libraries like Pandas [13], Numpy [14], Scikit-learn [15], Biopython [16], Gensim [17], Altair [18], and Streamlit. Tabular data sets (CSV or TSV) are internally imported as Pandas Data Frame structures. Archived data sets are first unpacked and then cleaned up using built-in Python libraries. If a FASTA data set is selected for proteomics or genomics, the web interface provides an option to calculate an additional physico-chemical data set. These properties are calculated with task-specific in–house algorithms by importing each FASTA sequence, one by one using Biopython, and then processing them. As of the time of writing, the additional data set created out of genomics FASTA data set contains three physico-chemical features — *Hydrogen Bond*, *Stacking Energy*, and *Solvation*. If an option to create an additional physico-chemical data set is selected for the proteomics FASTA data set, the data set consists of 43 physico-chemical features, e.g., *Molecular Weight*, *Isoelectric Point*, *Instability Index*, etc. The newly created data set incorporates the temporal dimension of the source data and can also be visualized as is. We integrated various methodologies into MOVIS to solve the embedding tasks. One of the central algorithms is the Word2Vec algorithm [19], [20] used to embed archived FASTA files into 100-dimensional vectors. Such embedding supports nucleotide- and amino-acid-based sequences. The dimension of the output vector was chosen empirically. Because each FASTA file may contain multiple sequences, we first embedded each sequence in a 100-dimensional vector and then averaged them to create one vector representing the FASTA file containing those sequences. This process results in a tabular data set containing the same number of rows as FASTA files in an archived dataset and 101 columns. Each column contains the temporal data, and the other 100 include the 100-dimensional vector embedding. The clustering step is presented with two clustering algorithms as options: KMeans [21] (distance-based) and OPTICS [22] (density-based). By having two different methods that achieve the same goal, different types of inductive principles are covered. The visualizations are created according to the nested model of visualization [23] and follow good data visualization practices [24]. That is to say, design considerations are taken to accommodate color blind users and enhance the accessibility of the visualizations to a broader audience. Other available data visualizations are reported in the supplementary material. Three different dimensionality reduction techniques are included to visualize clusters, namely PCA [25], [26], MDS [27], and t-SNE [28]. Although each of these methods aims to reduce the dimensionality of the data, their goal is different. Therefore, more choices provide a greater opportunity to distinguish patterns and anomalies in the data. The effect of choosing different dimensionality reduction techniques to visualize clusters of the same data set can be seen in Fig. 3. In this Figure, PCA was used for the upper, and MDS for the lower visualization.

## 4 Results

**Fig. 5:**
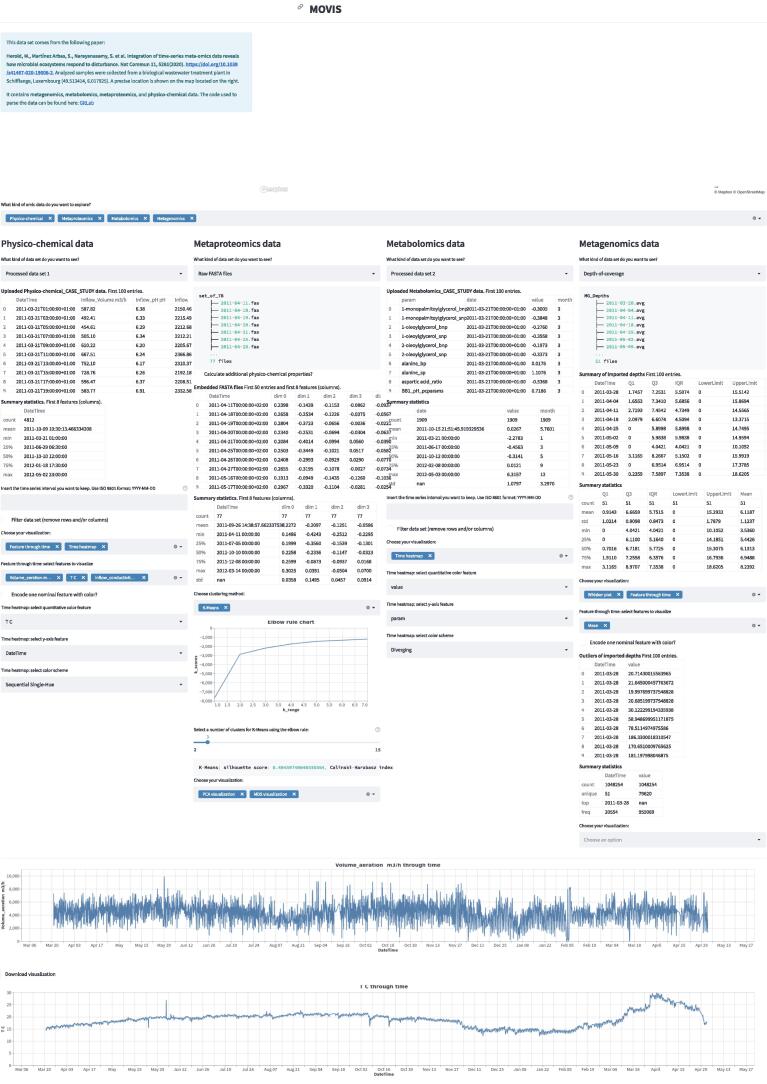
Case study overview, part 1. We can see all five parts of the tool’s workflow.

**Fig. 6:**
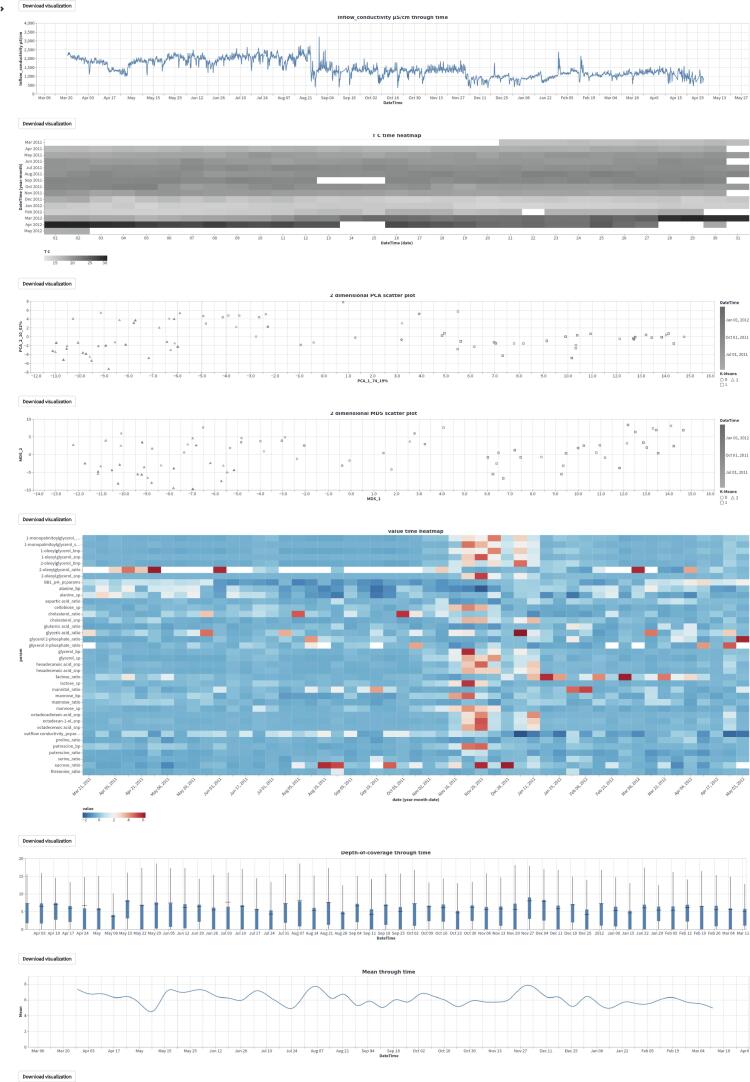
Case study overview, part 2. The second part of the overview is consisted only of the visualization canvas, with almost all visualization created for the case study.

To demonstrate the usability of MOVIS, we present a case study based on one of the available examples. The case study is also built-in into MOVIS as one of the options on the navigation sidebar. As presented in MOVIS, the case study overview can be seen in Fig. 5, Fig. 6.

### 4.1 Case study — Introduction

We focused on the built-in *Example 1* [29] (named as in MOVIS) that contains metagenomics, metaproteomics, metatranscriptomics, metabolomics, and physico-chemical data from the Biological WasteWater Treatment Plant (BWWTP). The data was collected in situ, at weekly intervals, and over 14 months. The end goal of this case study was to reveal if there were any niche types, and if there were, how did they respond to the substrate changes. To limit the scope of this use case, we proposed only using the functional aspects of the metabolomics data [29].

### 4.2 Case study — Main findings

Even though BWWTP operation is a controlled process, factors such as aeration cycles, seasonal changes in temperature, and composition of inflow wastewater fluctuate [30]. The physico-chemical factors may have a meaningful impact on population dynamics and linked process efficiency [31]. Therefore, the first step of our case study was to inspect relevant physico-chemical properties of the wastewater and determine major shifts, if any.

#### 4.2.1 Physico-Chemical data

**Fig. 7:**
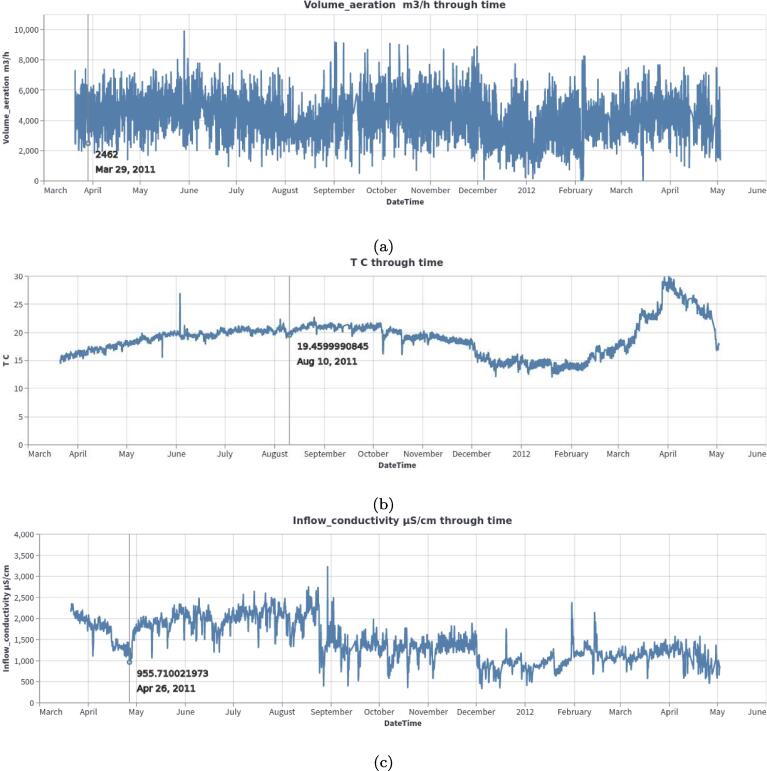
Relevant physico-chemical properties of the wastewater sludge. A seasonal pattern is present in the first figure, while we have more irregular readings in Figure (b) and Figure (c).

**Fig. 8:**
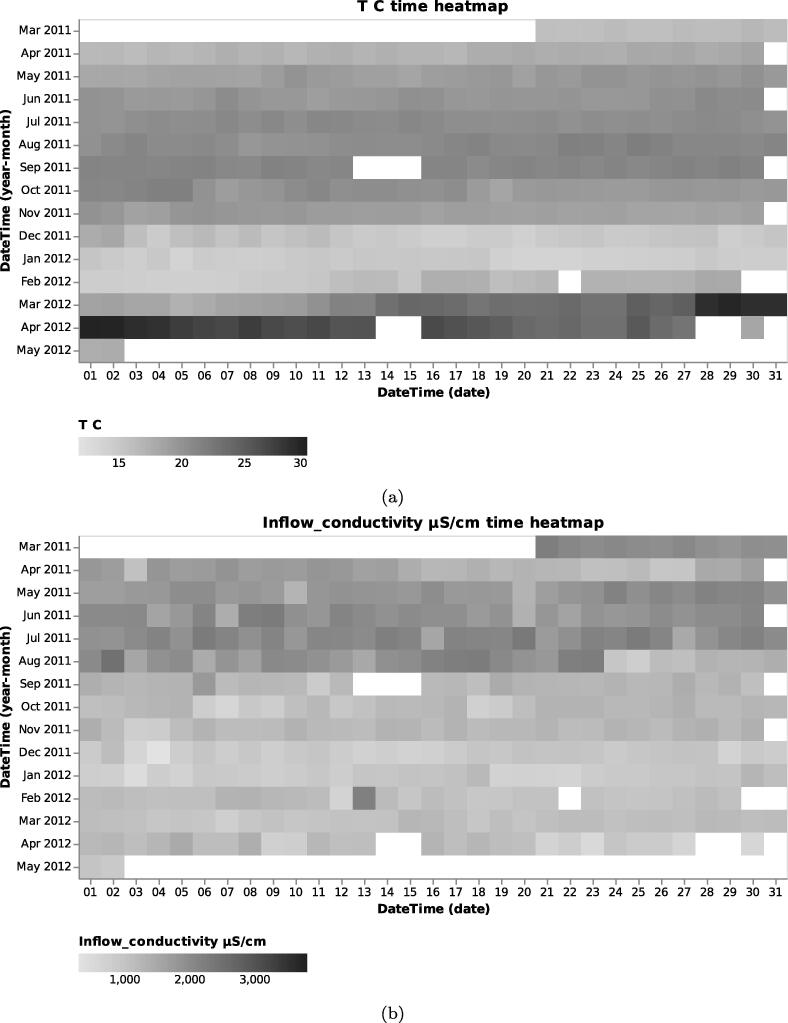
A closer inspection of temperature and inflow conductivity of the wastewater sludge. Figure (a) shows steady values on the upper half of the heatmap and a considerable variation on the lower half. Figure (b) shows a rapid transition from higher to lower conductivity values. The shift happens roughly after one-third of the sampling period.

To demonstrate the function and utility of MOVIS, we selected the *Physico-Chemical data* and, more specifically, the *Processed data set 1*. The selected data set contains 34 different physico-chemical properties with a 2-h sampling rate. The properties of interest for this case study are *Volume*_*aeration m3/h*, *T C* (Temperature in Celsius), and *Inflow*_*conductivity* μ*S/cm*. We visualized the properties of interest using MOVIS in Fig. 7, Fig. 7, Fig. 7, respectively. We chose *Feature through time* visualization to accomplish this. Even with a lot of noise present, Fig. 7a showed the shape of a sine curve with three distinct local maxima (around June 2011, November 2011, and April 2012) and two distinct local minima (around August 2011 and January 2012). Fig. 7, Fig. 7c had less noise, with the latter figure showing a distinct increase in temperature near the end of the sampling period. In order to further examine the irregular behavior of temperature and conductivity, we visualized them using *Time Heatmap* in addition to the existing chart types. The new visualizations are presented in Fig. 8, Fig. 8, respectively. Provided with more granularity, we were able to see a detailed picture of each property. Fig. 8a showed a steady increase in temperature that peaks in September of 2011 and then slightly decreases until the start of December 2011. Then, we have a rapid decrease in temperature that lasts until March 2012 followed by a rapid increase that peaks in the beginning of April 2012 with temperatures going as high as 29.62 °C. We efficiently inspected values by using the interactive tooltip feature. It appears upon the mouse hovering over the cells of interest. On the other hand, Fig. 8b showed high but steady values from the beginning of sampling and up until the last week of August 2011. After that, we found a swift decrease and stabilization of values that continues throughout the time series.

#### 4.2.2 Metaproteomics data

**Fig. 9:**
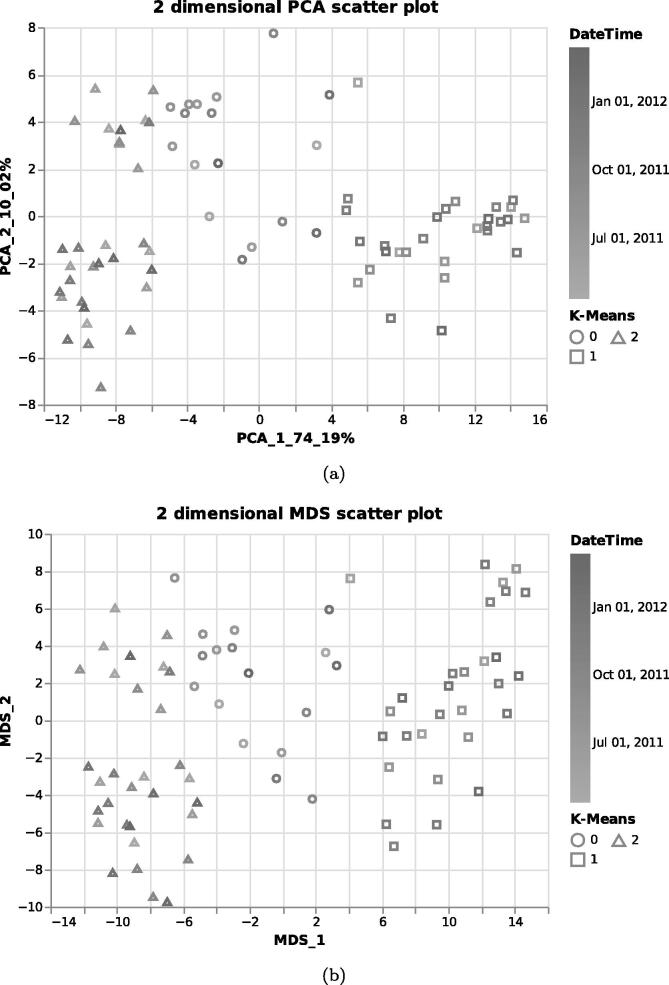
Clustered FASTA embeddings of the metaproteomics data set. Figure (a) used the PCA dimensionality reduction technique to visualize embedded data, while Figure (b) used the MDS technique.

Integrated meta-omics approaches hold the potential to resolve niches of microbial populations in situ [29]. Therefore, we shifted our focus to metaproteomics data to identify microbial clusters, if there were any, using only raw FASTA data. To ascertain the presence of microbial clusters, we selected *Metaproteomics data*, and then *Raw FASTA files*. When MOVIS completed embedding FASTA files, we selected the *K-Means* clustering method and using the *Elbow rule chart* we selected three as a number of clusters (centroids) for our clustering method. Then we inspected the evaluation window of the selected clustering method. With a silhouette score [32] of 0.495, we acknowledged that our method was successful, which was further corroborated by other available evaluation scores (e.g., The Davies-Bouldin index (DBI) [33], and The Calinski-Harabasz (CH) score [34]). Then we chose to visualize our data using two different dimensionality reduction techniques in order to determine which one gives a better visual outcome. The selection of *PCA visualization* and *MDS visualization* resulted in Fig. 9, Fig. 9, respectively. Both figures provided us with a visual way to evaluate chosen clustering method. As can be seen on both Fig. 9, Fig. 9, their upper-left corner showed a mixture of class-0 (circles) and class-2 (triangles), which indicated that K-Means had problems with clustering data embeddings that occupy that space. However, the clustering was successful since most data embeddings were placed in cloud points that have been determined to be in proximity and define a cluster. Inspecting the color gradient of the visualization marks allowed us to discover even more — samples clustered in the class-1 (rectangles) came in majority from the later time of the sampling period. The same could also be said for the class-2 samples, while class-0 samples came from a more dispersed sampling period. Mouse-hovering over each sample allowed us to determine the exact time that sample was collected. MOVIS also supports calculating amino-acid based physico-chemical properties of the metaproteomics data set, which could uncover an even more detailed picture of the underlying phenomena. For the sake of brevity, we did not select that option.

#### 4.2.3 Metabolomics data

**Fig. 10:**
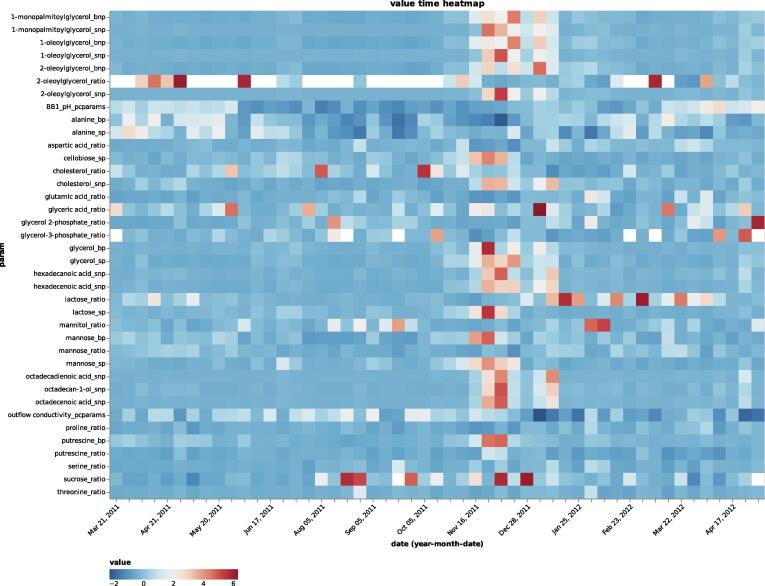
Metabolite and physico-chemical values over time. A major shift of multiple parameters can be clearly observed around November 2011. Important abbreviation: **bnp** — intracellular nonpolar metabolites, **bp** — intracellular polar metabolites, **ratio** — metabolite intracellular/extracellular ratio, **snp** — extracellular nonpolar metabolites, **sp** — extracellular polar metabolites.

Since a significant shift in substrates of the influent wastewater sludge can alter the community composition [35], we moved our focus to the *Metabolomics data set*, and more specifically the *Processed data set 2*. The selected data set is of composite nature, which means that it contains multi-omic information. Almost 95% of the data set represents metabolomics data, and the rest is physico-chemical data. Pre-combining omics data in such a fashion allows MOVIS to tap into the integrative aspect of the multi-omics nature. That aspect is planned but not yet directly available in MOVIS. Next, we selected *Time heatmap* visualization and chose feature named *value* as a *quantitative color feature*, *param* as a *y-axis feature*, and *Diverging* for the *color scheme*. Our selection resulted in Fig. 10. Further inspection of Fig. 10 revealed substrate shift happening from early to mid-November 2011 and early to mid-December 2011, with noticeably higher values in between. The substrate shift was defined by higher values of mainly non-polar metabolites, as well as polar metabolites, among which are putrescine and various disaccharides. After the end of December 2011, substrate levels normalized, and the community transitioned back to the pre-disturbance state.

#### 4.2.4 Metagenomics data

**Fig. 11:**
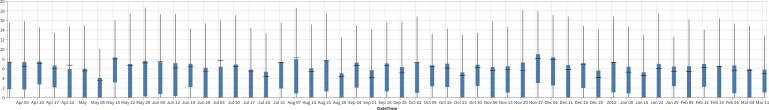
Metagenomics depth-of-coverage over time. A sinusoid wave formed by third quartile and upper limit values can be observed.

One way of estimating population abundance is by using metagenomic depth-of-coverage. Since MOVIS is not explicitly designed to work with meta-omics data, no taxa linking is currently enabled. However, by inspecting average depth-of-coverage values, we could get some insights into the overall population dynamics over time. Therefore, we selected *Metagenomics data*, and then *Depth-of-coverage*, which presented us with a directory hierarchy of the underlying data set. After MOVIS automatically calculated important statistical values of the data set in use, we visualized results using *Whisker plot*. The visualization mentioned earlier can be seen in Fig. 11. The third quartile (Q3) and upper limits form the shape of a sine curve with a period of around one month and a slight discrepancy around the beginning of November 2011. The discrepancy is caused by the increase of outliers (not shown in Fig. 11) while calculating statistical values. We then visualized *Mean* depth-of-coverage values using *Feature through time* visualization. However, that did not provide us with any new insight.

### 4.3 Case study — Conclusion

The simultaneous exploration of multi-metaomics data sets using MOVIS allowed us to uncover temporal patterns and discrepancies of one metaomic data set and efficiently connect them with other metaomic data sets. Furthermore, a swift visualization of sizable time-series multi-modal data sets revealed significant microbial clusters and temporal points of interest. Withal, we are now empowered further to analyze temporal points of interest with metaomic-specific tools and uncover metaomic-specific details.

## 5 Discussion

First and foremost, MOVIS is the only time-series omics data exploration tool that is able to generate publication-ready visualizations of the underlying data by following best practices for user interface and data visualization design. Second, since it was written in a procedural way, it allows quick extensions with minor modifications. For example, the adoption of a new omic-type data requires the addition of one high-level function to the data analysis core. Third, the data analysis core presupposes that the data is preprocessed, that is to say, quality controlled and filtered. While FASTA files may be directly used as input, specific data such as raw microarray-based data cannot be used directly as input. The complexity of each omics domain knowledge makes this a challenging problem. Fourth, to support data exploration and visual analytic tasks, we rely on direct data interaction for tabular data. This interactivity lays the foundation for solving visual analytic tasks [36]. Fifth, clear and concise data analysis guidelines benefit multi-omics time-series analyses. Indeed, further omics-wide guidelines, time-series-specific, and data-specific standards are required. Sixth and last, we plan to integrate and make available many more data sets, omic-types data, clustering algorithms, dimensionality reduction methods, and visualizations. Moreover, the implementation of many other embedding algorithms should provide users with ample choice to accommodate the varying nature of the underlying imported data. In this line of reasoning, we are open to requests to include more data in the tool. Currently, two sample data sets are already integrated and available for exploration [29], [37]. Since the scope of the data and its size can have a significant impact on computational performance, we also plan to adapt MOVIS to cloud computing.

## 6 Conclusion

MOVIS is created as a modular and easy-to-use solution that includes state-of-the-art libraries and models to import, embed, cluster, and visualize temporal omics data. We expect that the proposed MOVIS will be a valuable tool to complement and enhance traditional data exploration approaches for temporal omics data and offer further insights into the patterns and anomalies of any of the five available omics types and their potential combination. MOVIS currently supports genomics, transcriptomics, metabolomics, proteomics, physico-chemical data, and metaomic aspects of aforementioned omic types.

## Data availability

We provide MOVIS as a web service at https://movis.mathematik.uni-marburg.de/ and as a Docker container at https://hub.docker.com/r/aanzel/movis. The website version is free and open to all users, without any registration requirements. Source code, help, and documentation can be found at https://github.com/AAnzel/MOVIS. MOVIS is licensed under the GNU General Public License, Version 3.0, and can be manipulated, improved, and extended freely by any user.

## Author contributions statement

A.A. wrote the manuscript, designed and developed the tool. D.H. discussed the results and revised the manuscript. G.H. supervised the project, guided the tool development, proofread, and revised the manuscript. All authors read and approved the final manuscript.

## Funding

All authors are members of the MOSLA consortium, which has received funding from the Hessian Ministry for Science and the Arts (LOEWE).

## Declaration of Competing Interest

The authors declare that they have no known competing financial interests or personal relationships that could have appeared to influence the work reported in this paper.
