# Supplementary material for: MOVIS: A multi-omics software solution for multi-modal time-series clustering, embedding, and visualizing tasks
Source: Comput Struct Biotechnol J. 2022 Feb 22;20:1044–55. doi: 10.1016/j.csbj.2022.02.012 (PMC8886009; doi:10.1016/j.csbj.2022.02.012)
Supplement: Supplementary file 1 [file mmc1.pdf]

MOVIS: A Multi-Omics Software Solution for Multi-modal Time-Series Clustering, Embedding, and Visualizing Tasks

Aleksandar Anžel[a], Dominik Heider[a], Georges Hattab[a]

[a]*Department of Mathematics and Computer Science, University of Marburg, Hans-Meerwein-Strasse 6, Marburg, 35032, Hesse, Germany*

Keywords: genomics, transcriptomics, metabolomics, proteomics, physico-chemical, omics, multi-omics, time-series, temporal, multimodal, modality, clustering, embedding, visualization

Supplementary material

The supplementary material for this paper consists of various screenshots of MOVIS. Each screenshot depicts one or more essential functionalities or showcases a different part of the tool that the user experiences.

Email address: aleksandar.anzel@uni-marburg.de (Aleksandar Anžel)

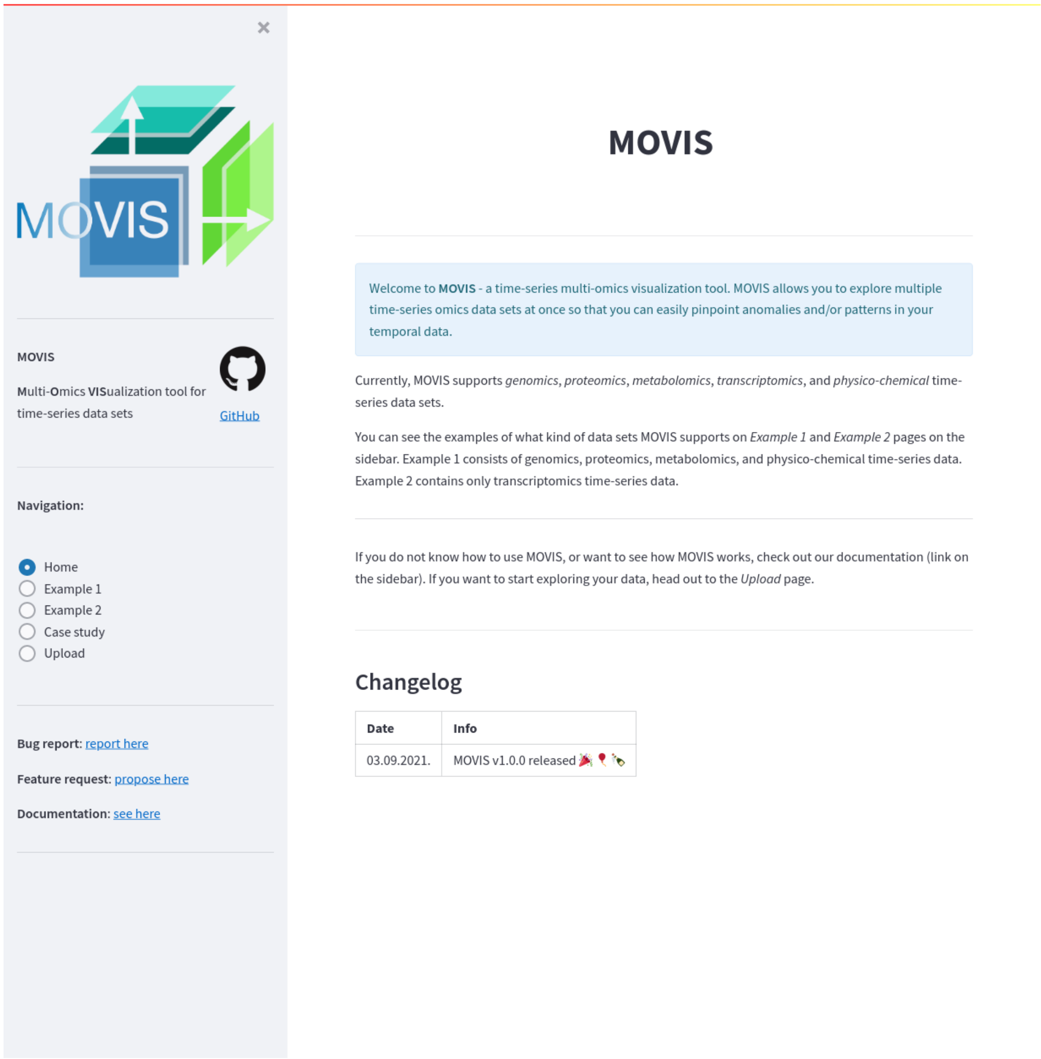

Figure 1: **The homepage.** The default page is a welcome page that briefly describes the tool and provides a changelog.

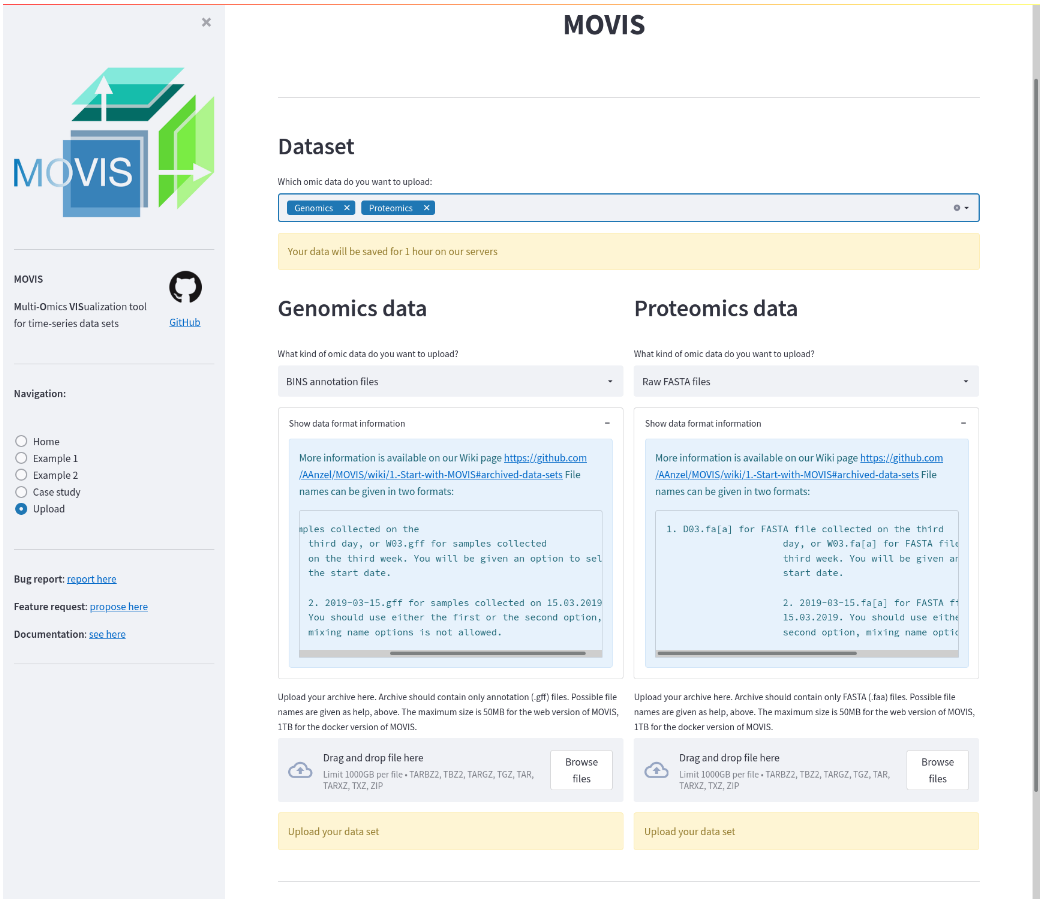

Figure 2: **The upload functionality.** Genomics and proteomics data are ready to be uploaded.

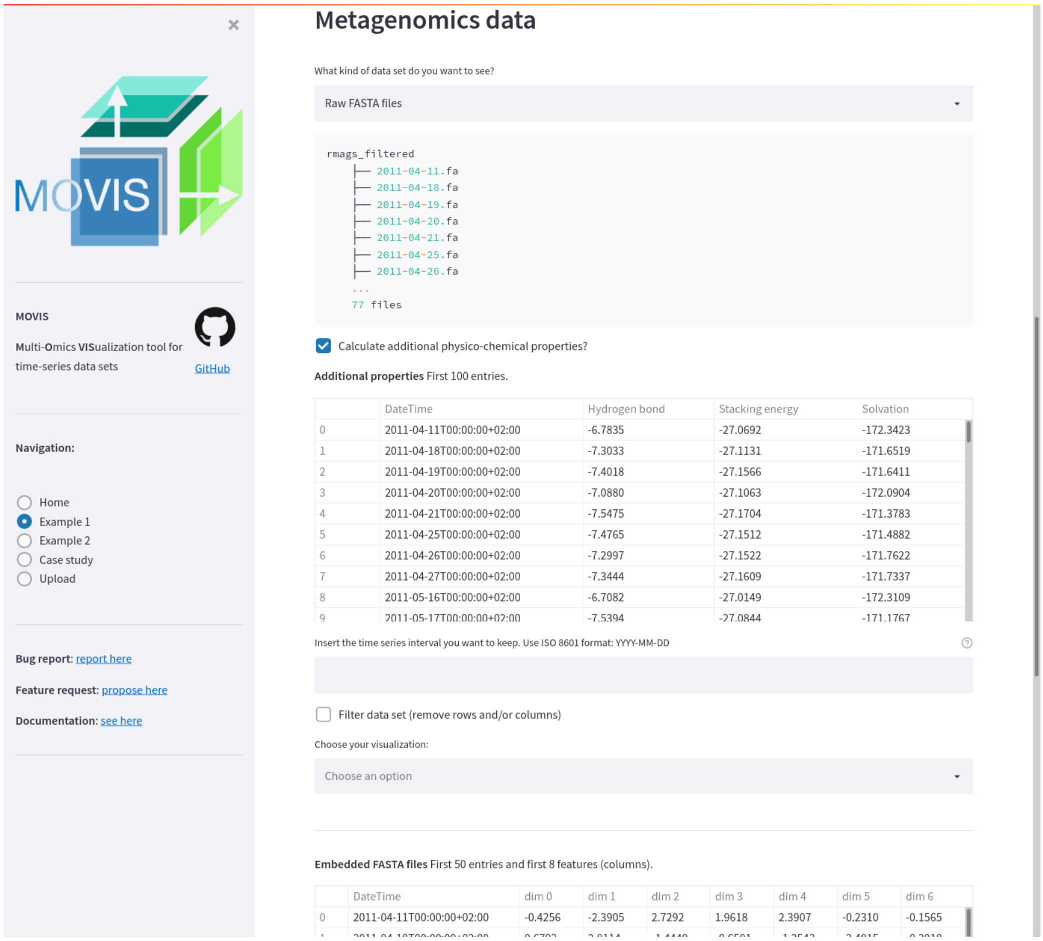

Figure 3: ***Example 1* data.** The screenshot shows additional physico-chemical properties calculated and displayed to the user. The first 50 entries of the embedded FASTA files are also displayed.

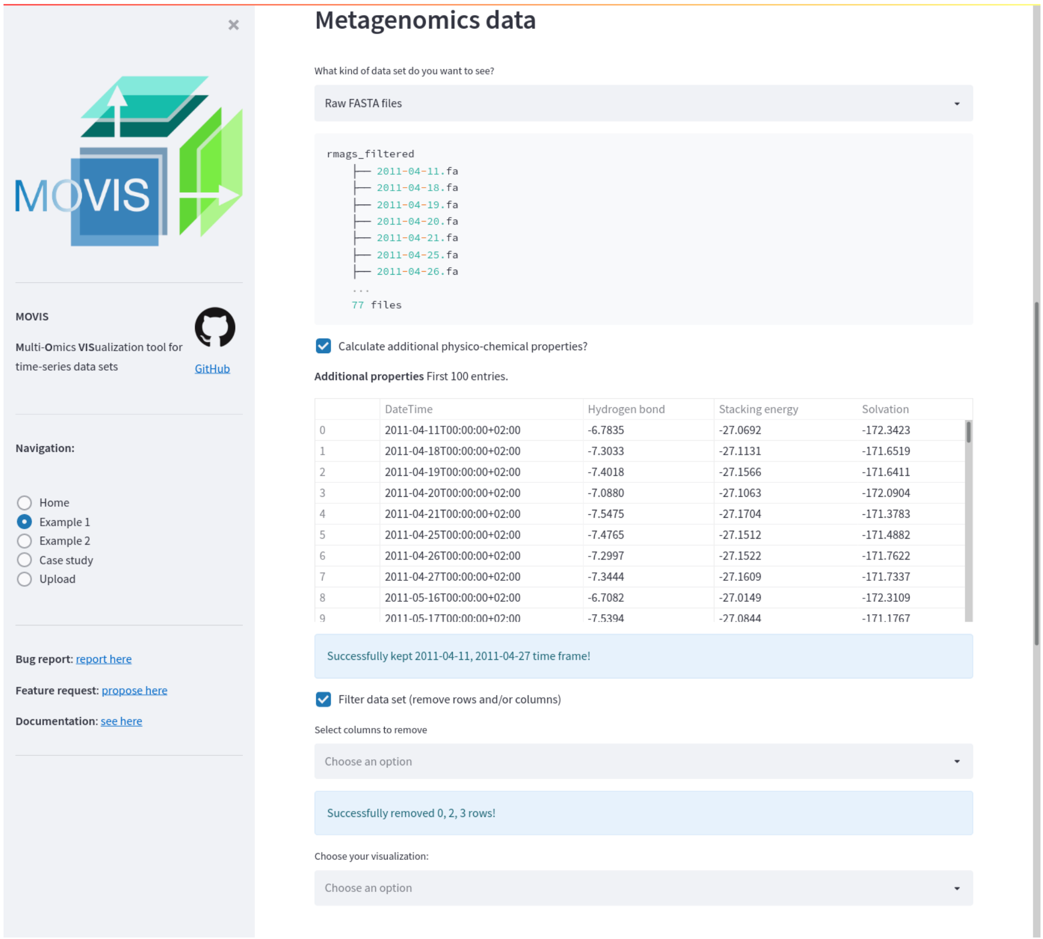

	DateTime	Hydrogen bond	Stacking energy	Solvation
0	2011-04-11T00:00:00+02:00	-6.7835	-27.0692	-172.3423
1	2011-04-18T00:00:00+02:00	-7.3033	-27.1131	-171.6519
2	2011-04-19T00:00:00+02:00	-7.4018	-27.1566	-171.6411
3	2011-04-20T00:00:00+02:00	-7.0880	-27.1063	-172.0904
4	2011-04-21T00:00:00+02:00	-7.5475	-27.1704	-171.3783
5	2011-04-25T00:00:00+02:00	-7.4765	-27.1512	-171.4882
6	2011-04-26T00:00:00+02:00	-7.2997	-27.1522	-171.7622
7	2011-04-27T00:00:00+02:00	-7.3444	-27.1609	-171.7337
8	2011-05-16T00:00:00+02:00	-6.7082	-27.0149	-172.3109
9	2011-05-17T00:00:00+02:00	-7.5394	-27.0844	-171.1767

Figure 4: **Genomics filtering on *Example 1* data.** A filtering step is added to slice the data from 2011-04-11 to 2011-04-27. Additionally, certain row numbers are omitted.

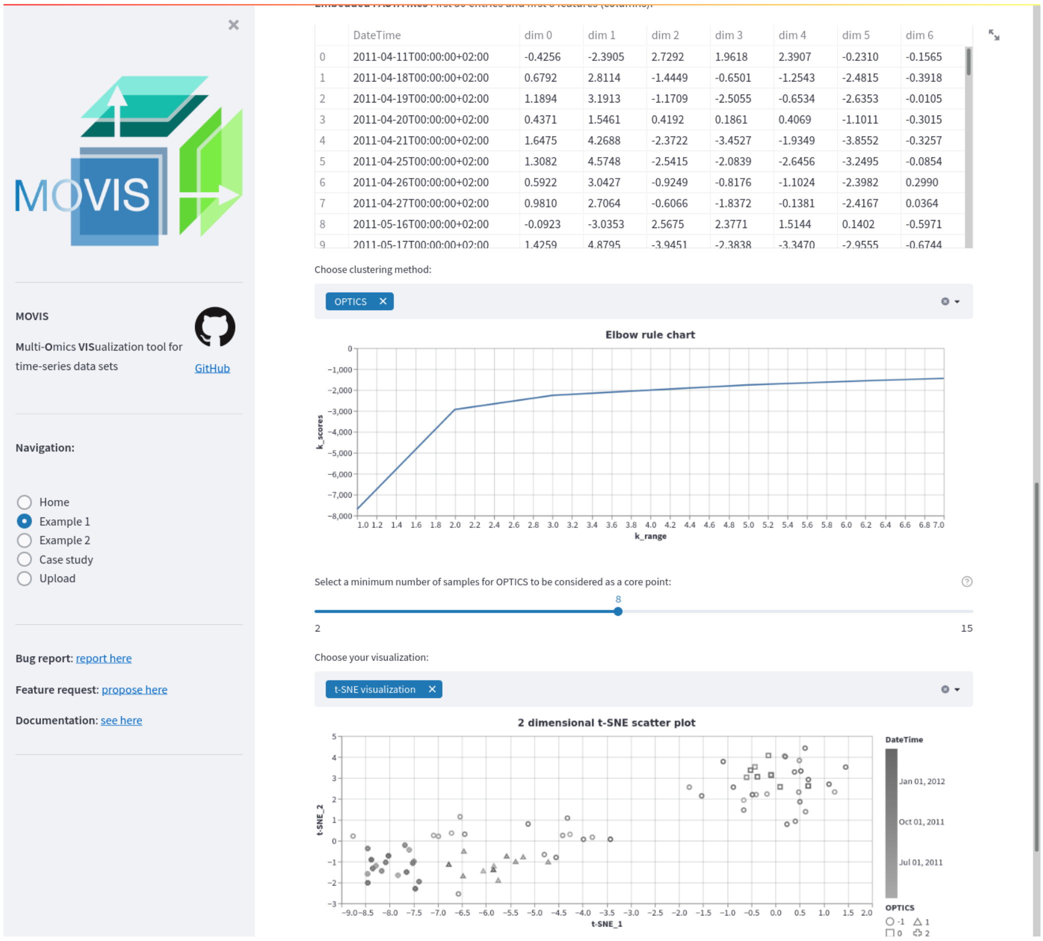

Figure 5: **Embedding for *Example 1* data.** The embedding of the resulting genomics data is displayed (50 first entries). A clustering method is applied: OPTICS. A threshold or a minimum number of samples for the method is set using a slider. To evaluate the results in the high dimensional space, the t-SNE visualization is chosen.

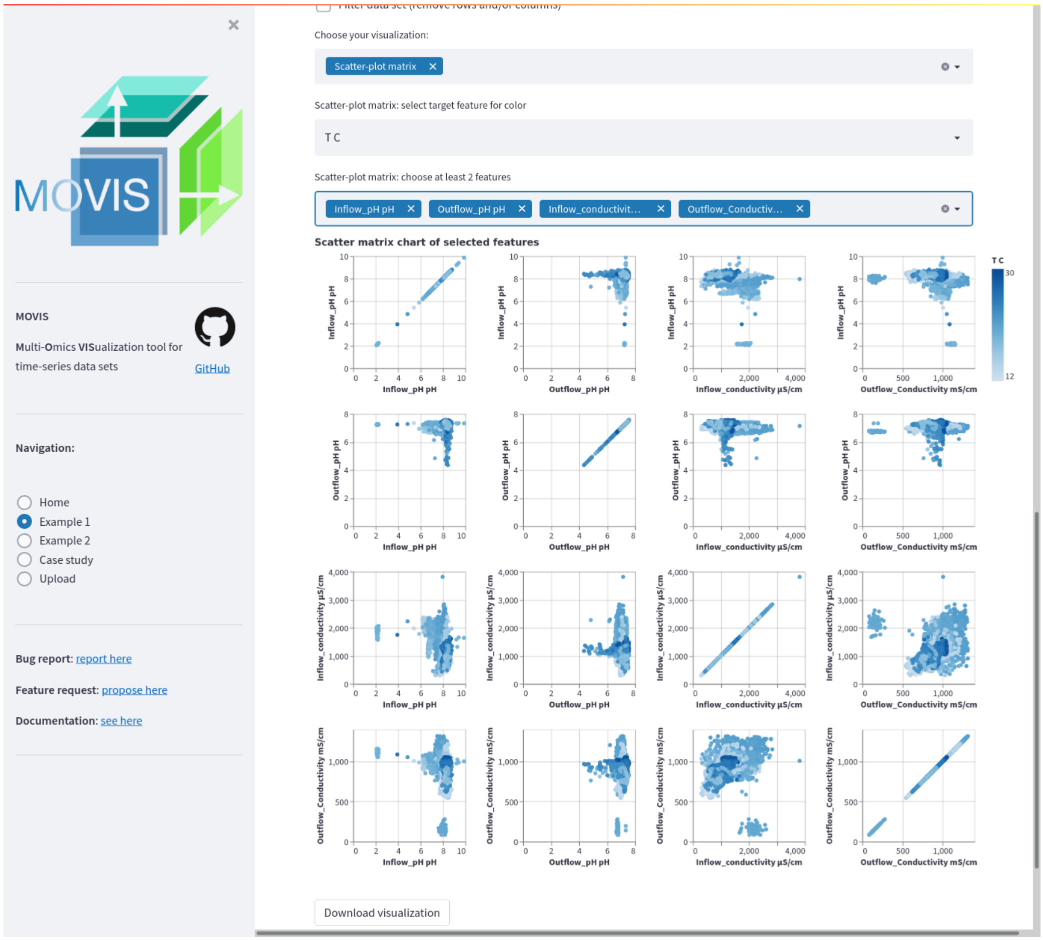

Figure 6: **Scatter plot matrix for selected features in physico-chemical data set.** Four features are selected: *Inflow_pH*, *Outflow_pH*, *Inflow_conductivity*, and *Outflow_conductivity*; totalling 16 possible scatter plot combinations which are displayed as a matrix using the small multiple idiom. A tooltip and a selection are available to investigate certain data points and their characteristics.

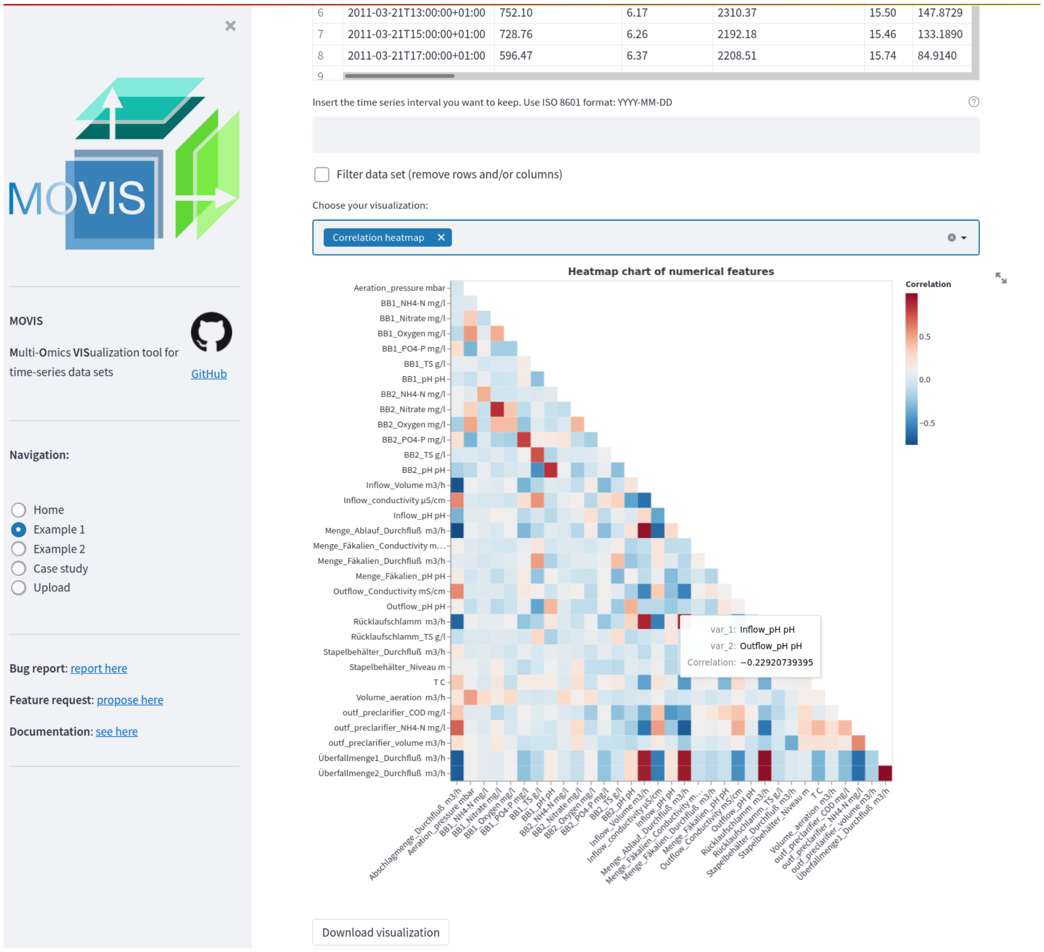

Figure 7: **Triangular heatmap of all features in the physico-chemical properties data set.** A diverging color scheme palette is selected to showcase low and high correlations with less emphasis on the mid-range values. A tooltip is available to investigate specific heatmap cells.

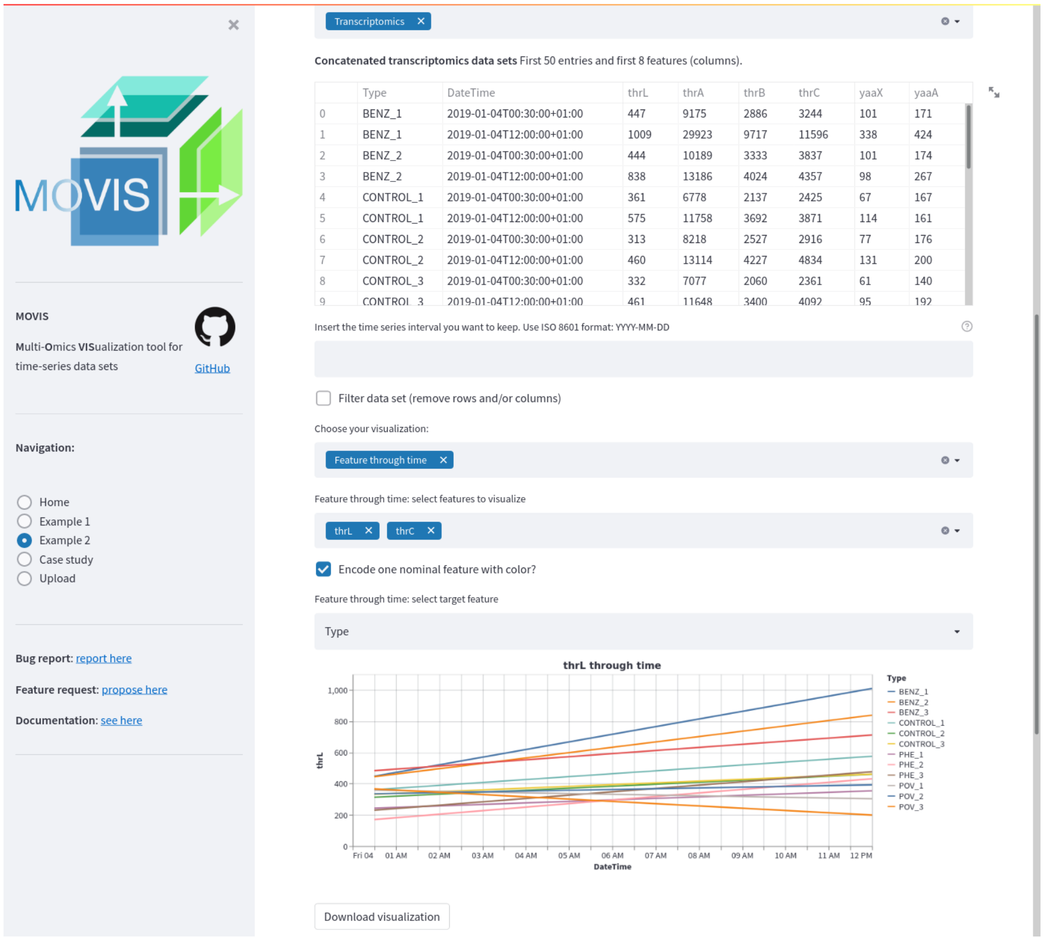

Figure 8: ***Example 2* transcriptomics data.** A time series of selected features is displayed as a line plot. Two features are selected: *thrL* and *thrC*. A categorical or nominal encoding is carried out for each of the 11 reported types. The *Tableau20* color palette is used.
